# Metabolic engineering of *Escherichia coli* for the production of riboflavin

**DOI:** 10.1186/s12934-014-0104-5

**Published:** 2014-07-16

**Authors:** Zhenquan Lin, Zhibo Xu, Yifan Li, Zhiwen Wang, Tao Chen, Xueming Zhao

**Affiliations:** 1 Department of Biochemical Engineering, School of Chemical Engineering and Technology, Tianjin University, Tianjin 300072, People’s Republic of China; 2 Key Laboratory of Systems Bioengineering, Ministry of Education, Tianjin University, Tianjin 300072, People’s Republic of China; 3 Collaborative Innovation Center of Chemical Science and Engineering (Tianjin), Tianjin University, Tianjin 300072, People’s Republic of China

## Abstract

**Background:**

Riboflavin (vitamin B_2_), the precursor of the flavin cofactors flavin mononucleotide (FMN) and flavin adenine dinucleotide (FAD), is used commercially as an animal feed supplement and food colorant. *E. coli* is a robust host for various genetic manipulations and has been employed for efficient production of biofuels, polymers, amino acids, and bulk chemicals. Thus, the aim of this study was to understand the metabolic capacity of *E. coli* for the riboflavin production by modification of central metabolism, riboflavin biosynthesis pathway and optimization of the fermentation conditions.

**Results:**

The basic producer RF01S, in which the riboflavin biosynthesis genes *ribABDEC* from *E. coli* were overexpressed under the control of the inducible trc promoter, could accumulate 229.1 mg/L of riboflavin. Further engineering was performed by examining the impact of expression of *zwf* (encodes glucose 6-phosphate dehydrogenase) and *gnd* (encodes 6-phosphogluconate dehydrogenase) from *Corynebacterium glutamicum* and *pgl* (encodes 6-phosphogluconolactonase) from *E. coli* on riboflavin production. Deleting *pgi* (encodes glucose-6-phosphate isomerase) and genes of Entner-Doudoroff (ED) pathway successfully redirected the carbon flux into the oxidative pentose phosphate pathway, and overexpressing the *acs* (encodes acetyl-CoA synthetase) reduced the acetate accumulation. These modifications increased riboflavin production to 585.2 mg/L. By further modulating the expression of *ribF* (encodes riboflavin kinase) for reducing the conversion of riboflavin to FMN in RF05S, the final engineering strain RF05S-M40 could produce 1036.1 mg/L riboflavin in LB medium at 37°C. After optimizing the fermentation conditions, strain RF05S-M40 produced 2702.8 mg/L riboflavin in the optimized semi-defined medium, which was a value nearly 12-fold higher than that of RF01S, with a yield of 137.5 mg riboflavin/g glucose.

**Conclusions:**

The engineered strain RF05S-M40 has the highest yield among all reported riboflavin production strains in shake flask culture. This work collectively demonstrates that *E. coli* has a potential to be a microbial cell factory for riboflavin bioproduction.

## Background

Riboflavin (vitamin B_2_) is the universal precursor of flavin mononucleotide (FMN) and flavin adenine dinucleotide (FAD), both of which act as hydrogen carriers and are essential for the activity of a wide variety of metabolic enzymes in higher eukaryotes [[1],[2]]. Riboflavin is synthesized by all plants, fungi and most bacteria, but not by higher animals including humans. So humans and animals must obtain riboflavin through dietary sources [[3]]. Traditionally, riboflavin was synthesized and produced by chemical procedures or microbial fermentation. Since chemical synthesis of riboflavin has many disadvantages such as high cost and energy wasting, the microbial fermentation method has been widely applied in industrial production.

**Figure 1:**
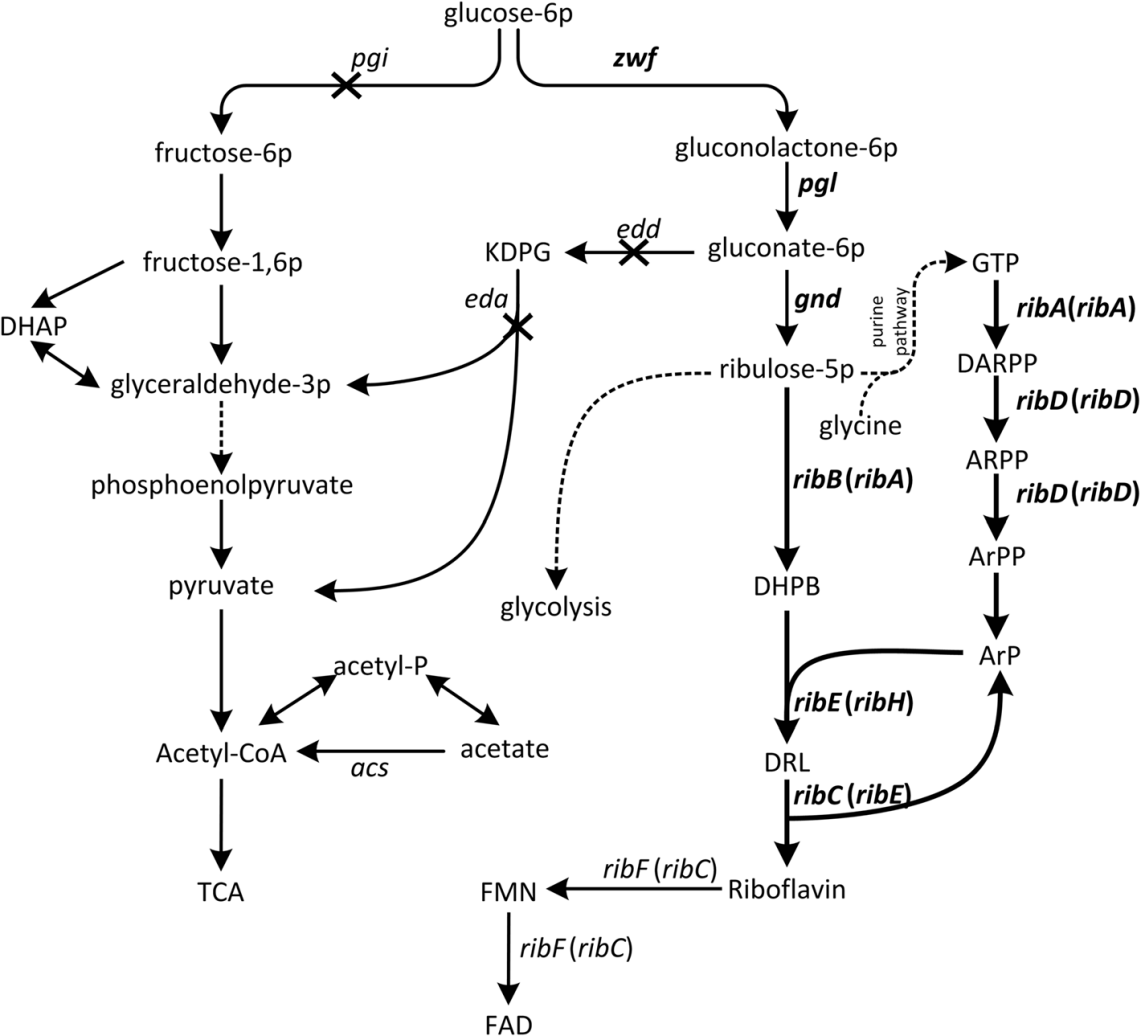
**Schematic overview of the relevant pathways of riboflavin production and engineering strategies for riboflavin production.***Bacillus subtilis* gene names were bracketed. Dashed lines indicate multiple enzymatic steps. The bold lines indicate the overexpression of the corresponding genes. The × indicates the deletion of the corresponding genes. Enzymes encoded by the genes shown are: *pgi*, phosphoglucose isomerase; *zwf*, glucose 6-phosphate dehydrogenase; *pgl*, 6-phosphogluconolactonase; *gnd*, 6-phosphogluconate dehydrogenase; *edd*, phosphogluconate dehydratase; *eda*, multifunctional 2-keto-3-deoxygluconate 6-phosphate aldolase and 2-keto-4-hydroxyglutarate aldolase and oxaloacetate decarboxylase; *ribA*, GTP cyclohydrolase II; *ribB*, 3,4-dihydroxy-2-butanone 4-phosphate synthase; *ribD*, fused diaminohydroxyphosphoribosylaminopyrimidine deaminase/5-amino-6-(5-phosphoribosylamino)uracil reductase; ribE, 6,7-dimethyl-8-ribityllumazine synthase; *ribC*, riboflavin synthase; *ribF*, bifunctional riboflavin kinase/FMN adenylyltransferase; *acs*, acetyl-CoA synthetase. The abbreviated metabolic intermediates are: KDPG, 2-keto-3-deoxy-6-phospho-D-gluconate; GTP, guanosine-triphosphate; DARPP, 2,5-diamino-6-ribosylamino-4(3H)-pyrimidinone 5'-phosphate; ARPP, 5-amino-6-(5′-phosphoribitylamino)uracil; ArPP, 5-amino-6-(5-phospho-D-ribitylamino)uracil; ArP, 5-amino-6-(D-ribitylamino)uracil; DHPB, 3,4-dihydroxy-2-butanone-4-P; DRL, 6,7-dimethyl-8-(1-D-ribityl)lumazine; FMN, riboflavin-5'-phosphate; FAD, flavin adenine dinucleotide; TCA, Tricarboxylic acid cycle.

Riboflavin biosynthesis has been studied in both gram-positive and gram-negative bacteria, extensively in *Bacillus subtilis* and *E. coli*. Riboflavin is synthesized from one molecule of GTP and two molecules of ribulose 5-phosphate (Ru-5-P) through a seven-step synthetic pathway, which is similar in all organisms (Figure 1) [[4]]. Riboflavin has been produced by the filamentous fungus *Ashbya gossypii*, the yeast *Candida famata*, and the bacterium *B. subtilis*, all of which have been developed by using a combined approach of classical mutagenesis and rational metabolic engineering [[3],[5]]. Thus, the genetic background of these strains was usually complicated and the mechanism of riboflavin overproduction was not fully elucidated.

In a series of previous reports, a numbers of conceivable strategies were carried out to develop *B. subtilis* as a riboflavin-producing strain after selection for resistance to different antimetabolites. In *B. subtilis*, the riboflavin production was increased by amplifying copies of *rib* operon or *ribA* gene [[6],[7]], or by enhancing the energy generation and reducing the maintenance metabolism [[8]–[10]]. Other efforts focused on modulating precursor metabolites supply by increasing the carbon flow through the pentose phosphate pathway (PP pathway) [[11]–[14]], by deregulating the gluconeogenesis flux via inactivating the *ccpN* [[15]], or by enhancing the flux of purine synthesis pathway [[16],[17]].

*E. coli* has been employed for efficient production of biofuels, amino acids, and bulk chemicals [[18]–[20]]. Although wild type *E. coli* does not accumulate riboflavin under natural conditions, it may be an efficient host for the production of riboflavin due to its clear genetic background, fast-growing, low maintenance metabolism, and the presence of convenient metabolic engineering tools. Thus, the aim of this study was to understand the metabolic capacity of *E. coli* for the production of riboflavin by modification of central metabolism, riboflavin biosynthesis pathway and optimization of the fermentation conditions.

In this study, the riboflavin synthetic pathway from *E. coli* and *B. subtilis* was constructed and compared in *E. coli* MG1655. Then, we examined the impact of overexpressing mutation type of *zwf* and *gnd* genes from *C. glutamicum* and *pgl* from *E. coli* on riboflavin production. Third, the glycolysis and ED pathway were modified to reroute carbon flux to the PP pathway for improving the riboflavin synthesis. Fourth, the acetate accumulation was reduced by overexpressing the *acs* gene. Finally, riboflavin production was significantly increased by modulating the expression of *ribF*. We also optimized the fermentation conditions for the riboflavin production. The final recombinant strain RF05S-M40 produced 2702.8 mg/L riboflavin with a yield of 137.5 mg riboflavin/g glucose under the optimized batch fermentation condition.

## Results and discussion

### Construction of riboflavin synthetic pathway

Previous researches indicated that overexpression of the genes of the riboflavin synthetic pathway contributed to the efficient production of riboflavin. In *B. subtilis*, introduction of multiple copies of *rib* operon into the chromosome resulted in improving the riboflavin production [[7]]. In *Pichia pastoris*, overexpression of the riboflavin biosynthetic pathway caused riboflavin production increased to 175 mg/L riboflavin although the wild type *P. pastoris* did not accumulate riboflavin [[21]]. In *C. famata*, overexpressing the gene related to the riboflavin production significantly increased riboflavin production [[22]].

**Figure 2:**
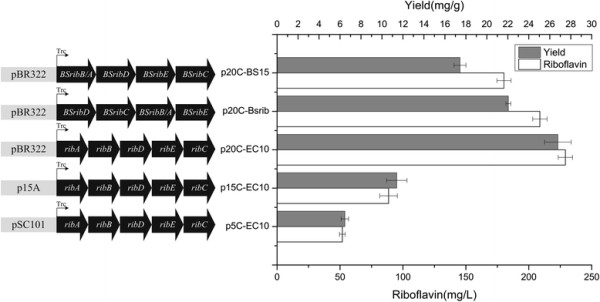
**Performance of riboflavin biosynthesis pathway with different plasmid replication origin and gene source.** Plasmid backbone p20C, p15C and p5C have pBR322, p15A and pSC101 replication origin, respectively. *ribA, ribB, ribD, ribE*, and *ribC* are genes cloned from *E. coli*; BSribA, BSribB, BSribD, BSribE, BSribC genes are cloned from *B. subtilis*. Trc, Trc promoter. Gray bars represent riboflavin titer (mg/L). Blank bars represent riboflavin yield (mg riboflavin/g-consumed glucose). Symbols represent the mean values for each parameter ± standard deviation of measurements from three independent experiments.

As shown in Figure 1, the genes of the riboflavin synthetic pathway (*ribA, ribB, ribD, ribE, ribC*) from *E. coli* were assembled into an artificial operon called EC10, under the control of the inducible trc promoter (Ptrc). Since the enzymatic activity of GTP cyclohydrolase II and 3, 4-dihydroxy-2-butanone 4-phosphate synthase played a critical role in riboflavin biosynthesis [[21]–[23]], the corresponding encoding genes *ribA* and *ribB* from *E. coli* were rearranged as the first and second genes in the operon. To test the dosage effect of the operon on riboflavin production, EC10 was inserted into plasmids with different copy numbers, generating p5C-EC10, p15C-EC10 and p20C-EC10, which have pSC101, p15A and pBR322 replication origin, respectively. Wild type *E. coli* MG1655, harboring p5C-EC10, p15C-EC10 and p20C-EC10, produced 50.5±1.2, 88.6±7.0 and 229.1±5.7 mg/L riboflavin (Figure 2), respectively. These results indicated that higher copy number of riboflavin synthetic genes was beneficial for riboflavin overproduction, which was in accordance with previously published results [[3],[7]].

The traditional gene names are different in *E. coli* and *B. subtilis* (Figure 1). For consistency, we use the *E. coli* gene names throughout. Thus, the *B. subtilis ribA*, *ribD*, *ribH*, *ribE* genes are renamed to *ribB*/*A*, *ribD*, *ribE*, *ribC*, respectively (Figure 1). To test the productivity of the riboflavin pathway from *B. subtilis*, plasmid p20C-BSrib was constructed by directly inserting the native *rib* operon from the genome of *B. subtilis* into p20C. MG1655 harboring p20C-BSrib produced 200.5±7.1 mg/L riboflavin (Figure 2). Moreover, to construct a synthetic operon devoid of native regulation, the riboflavin pathway genes from *B. subtilis* were individually amplified, rearranged according to the order in the metabolic pathway (BS*rib*(*B/A*)*DEC*), and assembled into p20C, creating plasmid p20C-BS15. *E. coli* MG1655 harboring p20C-BS15 produced 180.3±5.6 mg/L riboflavin (Figure 2), which was slightly lower than that of p20C-BSrib. Moreover, the activities of key enzymes of riboflavin synthesis, GTP cyclohydrolase II and riboflavin synthase in MG1655(p20C-BS15) were lower than that in MG1655(p20C-BSrib) (Table 1). This phenomenon may be due to that the internal promoters of native *rib* operon were removed after rearranging the gene order [[24]], which led to a decrease in the expression level of key genes *ribB/A* and *ribC*. Among all the tested *rib* gene expressing plasmids, p20C-EC10 showed the highest riboflavin productivity. Thus, strain RF01S (MG1655 harboring p20C-EC10) was selected for further engineering.

**Table 1:** Activities of GTP cyclohydrolase II and Riboflavin synthase

**Strain/Plasmid**	**Enzyme activities (nmol/min/mg protein)**	
	**GTP cyclohydrolase II**^**a**^	**Riboflavin synthase**^**b**^
*E. coli* MG1655/p20C-EC10	8.6±0.4	14.3±1.6
*E. coli* MG1655/p20C-BSrib	6.4±0.3	10.9±0.4
*E. coli* MG1655/p20C-BS15	6.3±0.2	9.6±0.4

^a^One unit of enzyme activity catalyzes the formation of 1 nmol of 2,5-diamino-6-ribosylamino-4(3H)-pyrimidinone 5'-phosphate per min.

^b^One unit of enzyme activity catalyzes the formation of 1 nmol of riboflavin per min.

### Effects of *zwf*, *gnd*, and *pgl* overexpression on riboflavin production

Ribulose 5-phosphate is an important precursor for riboflavin biosynthesis. Overexpressing the genes of oxidative PP pathway increased the ribulose 5-phosphate supply and improved riboflavin production by 31% in *B. subtilis* [[13]]. Mutant genes *zwf*^***(A243T)***^ [[25]] and *gnd*^***(S361F)***^ [[26]] from *C. glutamicum*, which encode glucose 6-phosphate dehydrogenase and 6-phosphogluconate dehydrogenase with removal of feedback inhibition, were co-overexpressed by introducing plasmid p15Trc*-zg* into RF01S. As shown in Table 2, the enzyme activities of glucose 6-phosphate dehydrogenase and 6-phosphogluconate dehydrogenase were significantly increased. However, the resulting strain showed only a slight increase in riboflavin titer and yield, compared to that harboring p15Trc (Table 2). Due to the low endogenous activity of 6-phosphogluconolactonase in *E. coli* [[27],[28]], we further overexpressed *pgl* (encoding 6-phosphogluconolactonase) from *E. coli* along with *zwf* and *gnd* by transforming p15Trc*-zgp* into RF01S, which led to an increase of 18.9% and 38.7% in riboflavin titer and yield, compared to that harboring p15Trc (Table 2). However, compared to strain RF01S, overexpressing PP pathway genes did not significantly increase the riboflavin production, possibly because of the complex regulation of central metabolic pathway [[29]] and a high metabolic burden of heterologous protein production [[30]].

**Table 2:** Effect of overexpressing pentose phosphate pathway genes on riboflavin production and enzyme activities

**Strain/plasmid**	**OD600**^**a**^	**Riboflavin (mg/L)**	**Riboflavin yield (mg/g)**^**b**^	**Enzyme activity (nmol/min/mg protein)**^**c**^	
				**G6PDH**^**d**^	**6PGDH**^**d**^
RF01S/p15Trc	3.8±0.1	192.1±7.3	23.0±0.9	111.5±11.5	119.1±8.4
RF01S/p15Trc-*zg*	3.0±0.1	197.4±9.2	24.2±1.4	203.6±18.5	174.7±14.2
RF01S/p15Trc-*zgp*	3.1±0.1	228.4±0.9	31.9±1.3	199.0±20.0	177.1±17.6

^a^OD_600_ is the final optical density of the culture.

^b^mg/g: mg/g-consumed glucose.

Batch cultures of recombinant strains were carried out in LB medium containing 10 g/L glucose at 37°C as described in methods. Standard deviations were calculated from the results of three independent experiments.

^c^One unit of the enzyme activity was defined as the amount catalyzing the formation of 1 nmol of NADPH per min. Results are the mean values of three measurements.

^d^6PGDH, 6-phosphogluconate dehydrogenase; G6PDH, glucose 6-phosphate dehydrogenase.

### Disruption of *pgi* for improving riboflavin production

Based on the results of overexpressing the genes of PP pathway, the strategy of eliminating competitive pathway for redirecting the carbon flux into the oxidative PP pathway was employed to improve the riboflavin production. The disruption of *pgi* encoding phosphoglucose isomerase resulted in a blocked entry into glycolysis and compelled glucose 6-phosphate to be metabolized exclusively through PP pathway [[31]]. Thus, we disrupted *pgi* in MG1655, creating RF02. RF02 harboring p20C-EC10 (RF02S) accumulated 394.8±19.6 mg/L riboflavin, which was 72.4% higher than that of the reference strain RF01S (Table 3). In accord with previous studies, the *Δpgi* mutant strains showed strongly reduced glucose uptake rates and maximum specific growth rate [[32]]. One possible reason for this phenomenon was that the *Δpgi* mutation would result in high glucose 6-phosphate pool and led to the rapid degradation of *ptsG* mRNA, an important member of the PTS transport systems, which would decrease the glucose uptake capacity [[33]]. Moreover, the *Δpgi* mutation also diminishes the supply of PEP needed for PTS-mediated uptake of glucose and decreases the growth rate [[34]].

**Table 3:** Riboflavin production of the various strains constructed in the study

**Strain**	**OD600**^**a**^	**Biomass (g/L)**^**b**^	**Riboflavin (mg/L)**	**Riboflavin yield (mg/g)**^**c**^	**Specific productivity (mg-RF/g-DCW/h)**
RF02S	5.4±0.3	2.0±0.1	394.8±9.6	39.9±1.1	5.1±0.2
RF03S	5.2±0.4	1.9±0.1	559.9±8.9	59.9±0.8	8.2±0.3
RF05S	4.9±0.2	1.8±0.1	585.2±13.6	59.3±1.3	8.6±0.2

^a^OD_600_ is the final optical density of the culture.

^b^The average cell dry weight for all of the strains was 0.38 g/L per optical-density (OD600) unit of culture.

^c^mg/g: mg riboflavin/g-consumed glucose.

Batch cultures of recombinant strains were carried out in LBG medium (LB with 10 g/L glucose) at 37°C as described in methods. Standard deviations were calculated from the results of three independent experiments.

### Disruption of *edd* and *eda* for improving riboflavin production

The ED pathway has been shown to be inactive with glucose as the carbon source in wild-type *E. coli* [[35]], but it was activated in the *pgi* mutant strain [[31]]. Previous studies indicated that glucose was mainly metabolized through PP pathway and partially through ED pathway after *pgi* was disrupted [[36]].

The *edd* and *eda* transcription levels in the *Δpgi* strain RF02 were compared with parent strain MG1655 through RT-qPCR analysis. Consistent with previously research [[37]], the transcript abundance of *edd* and *eda* increased 2.21-fold and 2.36-fold in RF02, respectively, which indicated that ED pathway was up-regulated in *Δpgi* strain. Thus, *edd* and *eda* were disrupted in RF02 for further increasing the flux from 6-phosphogluconate to Ru-5-P, resulting in strain RF03. RF03 harboring p20C-EC10 (strain RF03S) produced 559.88±8.99 mg/L riboflavin, which increased by 41.8% compared to that of RF02S (Table 3). These results suggested that further increasing riboflavin precursors can be achieved via disrupting ED pathway in the *pgi* mutation strain.

### Effect of the *acs* promoter insertion on riboflavin production

Acetate, which led to growth retardation, was the main byproduct of these recombinant strains. Thus, the strategy of overexpressing the *acs* gene encoding acetyl-CoA synthetase was selected to reduce acetate secretion. The trc promoter was inserted in the upstream of *acs* gene in RF03, resulting in strain RF05. RT-qPCR analysis showed that the expression of *acs* gene in RF05S (RF05 harboring p20C-EC10) increased 4.80-fold compared with RF03S, indicating successful overexpression of *acs* gene. RF05S produced 585.2±13.6 mg/L riboflavin with a yield of 59.30 mg/g glucose, which did not change significantly compared to RF03S (Table 3). However, the acetate production decreased from 4.0 g/l to 1.5 g/L in strain RF05S, which was in accordance with the results presented by Lin et al. [[38]].

### Modulating the expression *ribF* for enhanced riboflavin production

According to previous studies, introducing a mutation in *ribC* gene (encoding flavokinase/flavin adenine dinucleotide synthetase) in *B. subtilis* could reduce enzymatic activity and resulted in riboflavin overproduction in the engineering strains [[39]]. The *ribC* mutation of *B. subtilis* also reduced the synthesis of FMN, which is a negative regulatory factor of *rib* operon transcription [[40]]. In *E. coli*, these enzymes were encoded by *ribF*, which is an essential gene for strain growth [[41]]. Therefore, we speculate that reducing the expression level *ribF* might reduce the conversion of riboflavin to FMN/FAD and increase riboflavin production.

**Figure 3:**
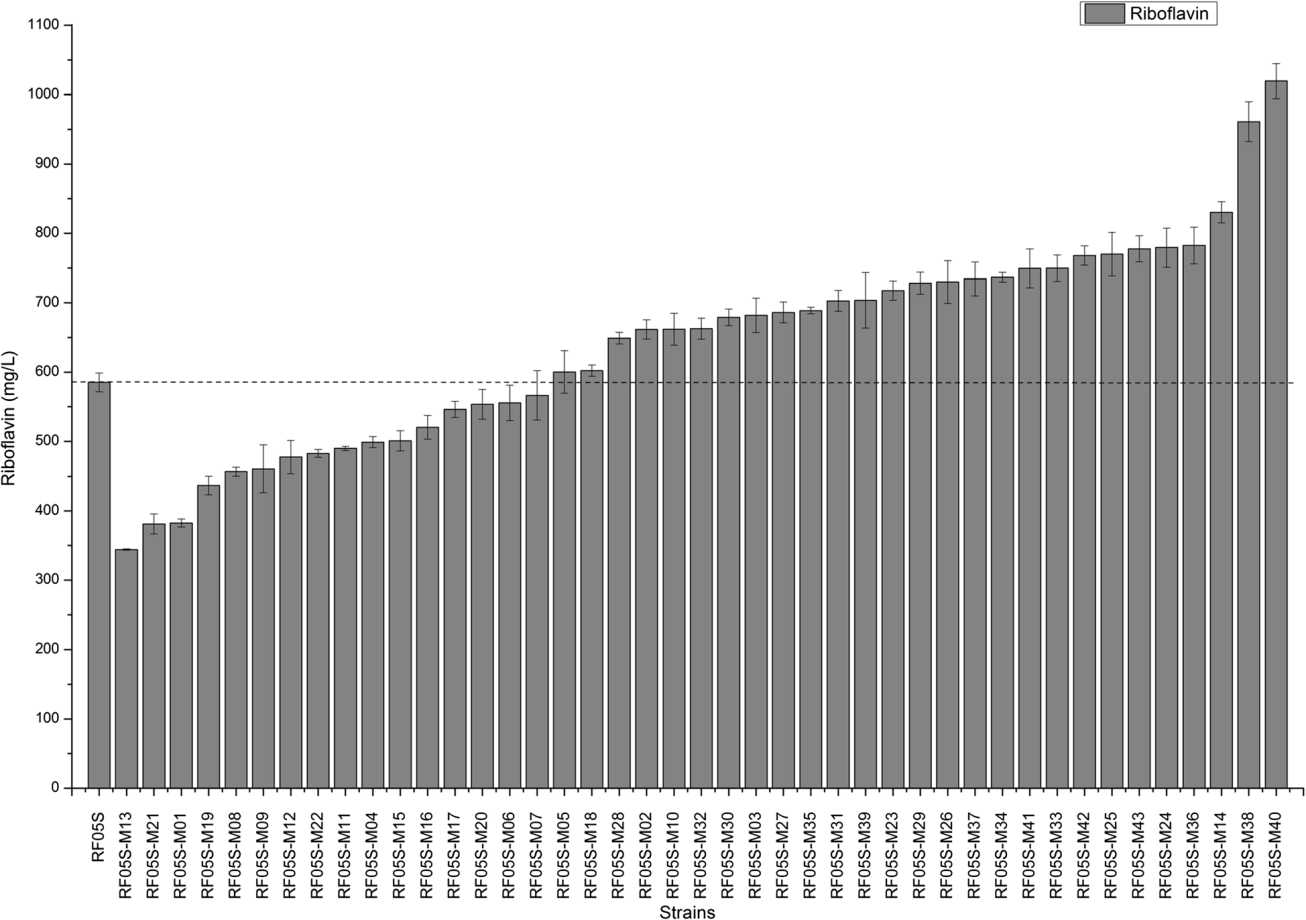
**Relevant riboflavin production of various RF05S mutants after fine-tuning ribF expression.** Strains were cultured in LB media with 10 g/L glucose at 37°C. Standard deviations were calculated from the results of three independent experiments.

**Figure 4:**
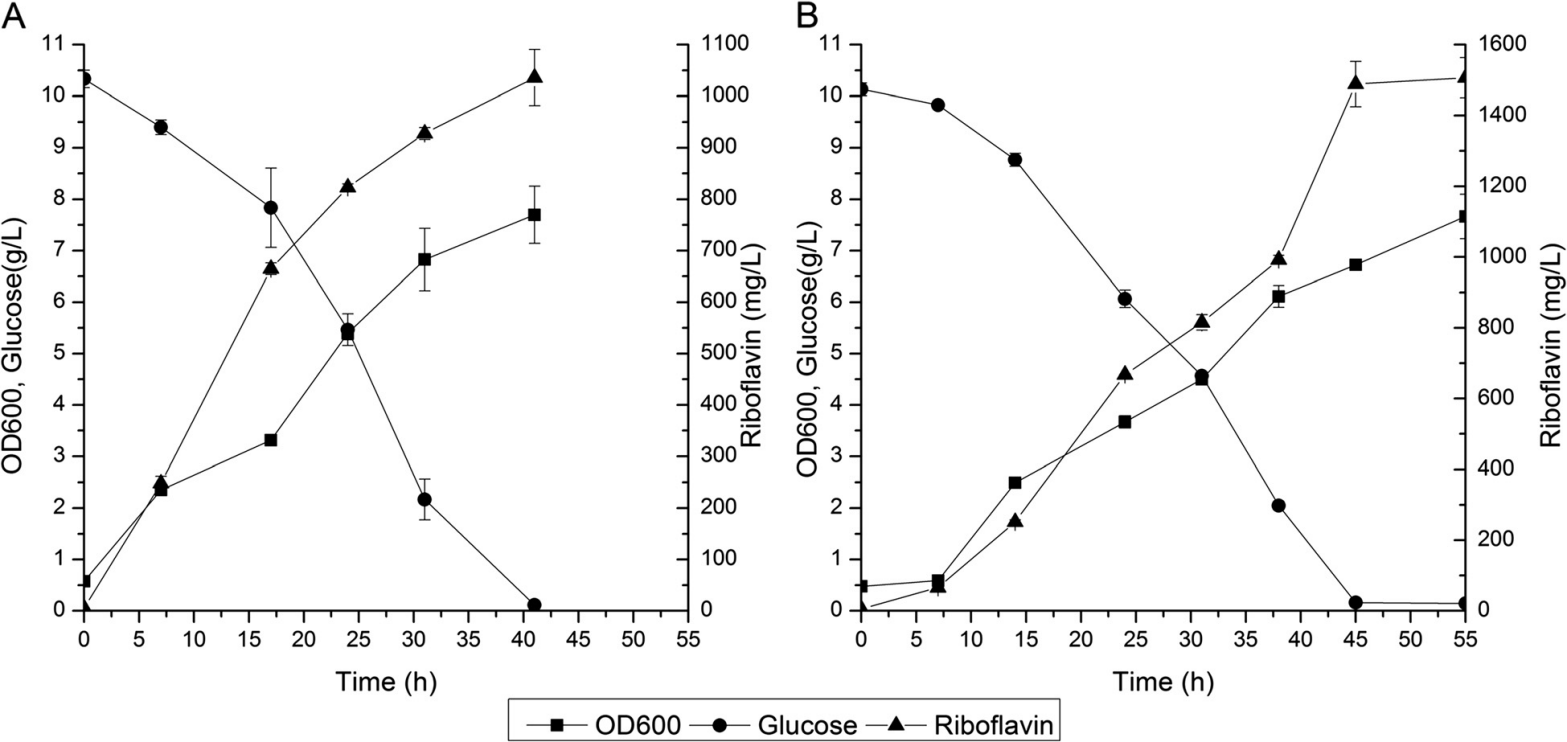
**Time-course profiles of riboflavin production, biomass accumulation and glucose consumption of RF05S-M40 in different temperatures.** Strains were cultured in LB media at 37°C **(A)** and 31°C **(B)**. Symbols represent the mean values for each parameter ± standard deviation of measurements from three independent experiments.

To modulate the expression of *ribF*, we designed a library of RBS sequences with varied strengths weaker than that of the native *ribF* RBS using RBS calculator [[42]]. Then, the native RBS of *ribF* on the chromosome of RF05S was replaced with the designed RBS library using the method described in methods. Forty three mutants were selected and transformed with p20C-EC10 to screen for enhanced riboflavin producer. Riboflavin production of the selected strains was measured after 48 h cultivation in LB medium with 10 g/L glucose at 37°C in flasks. The majority of the selected strains exhibited increased riboflavin production compared with the parent strain (Figure 3), which demonstrated that modulating *ribF* expression could efficiently increase riboflavin production. Strain RF05S-M40 produced 1036.1±54.6 mg/L riboflavin, which was the highest among all selected strains, and was 77.0% higher than that of RF05S. Representative profiles of riboflavin production, biomass accumulation and glucose consumption for the strain RF05S-M40 were shown in Figure 4A. Flavokinase activity in cell-free extracts of RF05S was about 1.45±0.09 nmol/min/mg protein, while that of RF05S-M40 was about 0.94±0.04 nmol/min/mg protein, indicating that the expression level of flavokinase was decreased by modulating the RBS of *ribF* and this reduced expression contributed much to riboflavin overproduction.

### Optimization of fermentation conditions for riboflavin production

To optimize fermentation condition, we investigated the effect of different culture temperatures on riboflavin production. Changing the fermentation temperature from 37°C to 31°C increased riboflavin titer to 1507.0±56.8 mg/L from 1036.1±54.6 mg/L (Figure 4B). Many factors may have contributed to the enhanced production. For example, growth temperature was reported to influence not only recombinant gene expression and protein folding, but also the global metabolism of the strains [[43]]. In addition, low growth temperature was reported to stabilize the recombinant proteins and improve the plasmid stability [[44]].

We further evaluated the riboflavin production of RF05S-M40 in semi-synthetic medium. Two different semi-synthetic medium formulations, M9 minimal medium and MS medium, were tested with different concentration of yeast extract at 37°C or 31°C (Table 4). When cells were cultivated in M9 minimal medium with 1 g/L yeast extract at 37°C, cell growth was severely hampered (OD 600 of 4), leading to a titer of 271.8±4.9 mg/L riboflavin (Table 4). Switching to another defined semi-synthetic medium (MS medium) led to much higher titers (Table 4). Using MS medium with 5 g/L yeast extract as culture medium exhibited the highest productivity in shake flask culture at 31°C. Optimization of the composition of MS medium further increased riboflavin production. Detailed composition of the optimized MS medium and riboflavin production profile were shown in Additional file 1: Table S2. Statistical analysis indicated that the adding of glycine in the medium contributed significantly to riboflavin overproduction. The reason for the increase in the riboflavin production might be that the supplement of glycine, which is an important purine precursor and is incorporated into the riboflavin molecule, enhanced the availability of GTP and thus improved the riboflavin production [[45]]. Similar phenomenon has been reported in *A. gossypii* and *C. famata* [[46]].

**Table 4:** Riboflavin production of RF05S-M40 in different cultural conditions

**Medium**	**Glucose (g/L)**	**Temperature (**°**C)**	**Yeast extract (g/L)**	**OD600**^**a**^	**Biomass**^**b**^**(g/L)**	**Riboflavin (mg/L)**	**Riboflavin yield (mg/g)**^**c**^	**Specific productivity (mg-RF/g-DCW/h)**
M9	10^d^	37	1	4.1±0.2	1.6±0.1	271.8±4.8	66.4±8.6	3.6±0.2
M9		37	3	7.0±0.1	2.7±0.1	289.5±7.9	25.2±0.8	2.3±0.1
M9		37	5	6.8±0.1	2.7±0.1	532.4±24.9	50.7±0.6	4.3±0.2
MS		37	1	6.4±0.1	2.4±0.1	702.4±17.4	66.0±0.7	6.1±0.2
MS		37	3	6.3±0.2	2.4±0.1	811.2±43.8	79.7±2.3	7.1±0.5
MS		37	5	7.7±0.3	2.9±0.1	847.4±38.0	82.7±3.2	6.1±0.4
M9		31	5	7.7±0.2	2.9±0.1	892.5±23.1	84.3±3.8	6.4±0.3
MS		31	1	6.5±0.2	2.1±0.1	577.6±11.4	66.5±1.9	4.9±0.2
MS		31	3	7.3±0.2	2.8±0.1	907.7±12.8	93.1±2.3	6.8±0.2
MS		31	5	7.6±0.2	2.9±0.1	1204.1±40.4	120.6±5.4	8.7±0.4

^a^OD_600_ was the final optical density of the culture.

^b^The average cell dry weight for all of the strains was 0.38 g/L per optical-density (OD600) unit of culture.

^c^mg/g: mg riboflavin/g-consumed glucose.

^d^Batch cultures of RF05S-M40 was carried out in modified M9 or MS containing 10 g/L glucose at 31°C or 37°C. Standard deviations were calculated from the results of three independent experiments.

**Figure 5:**
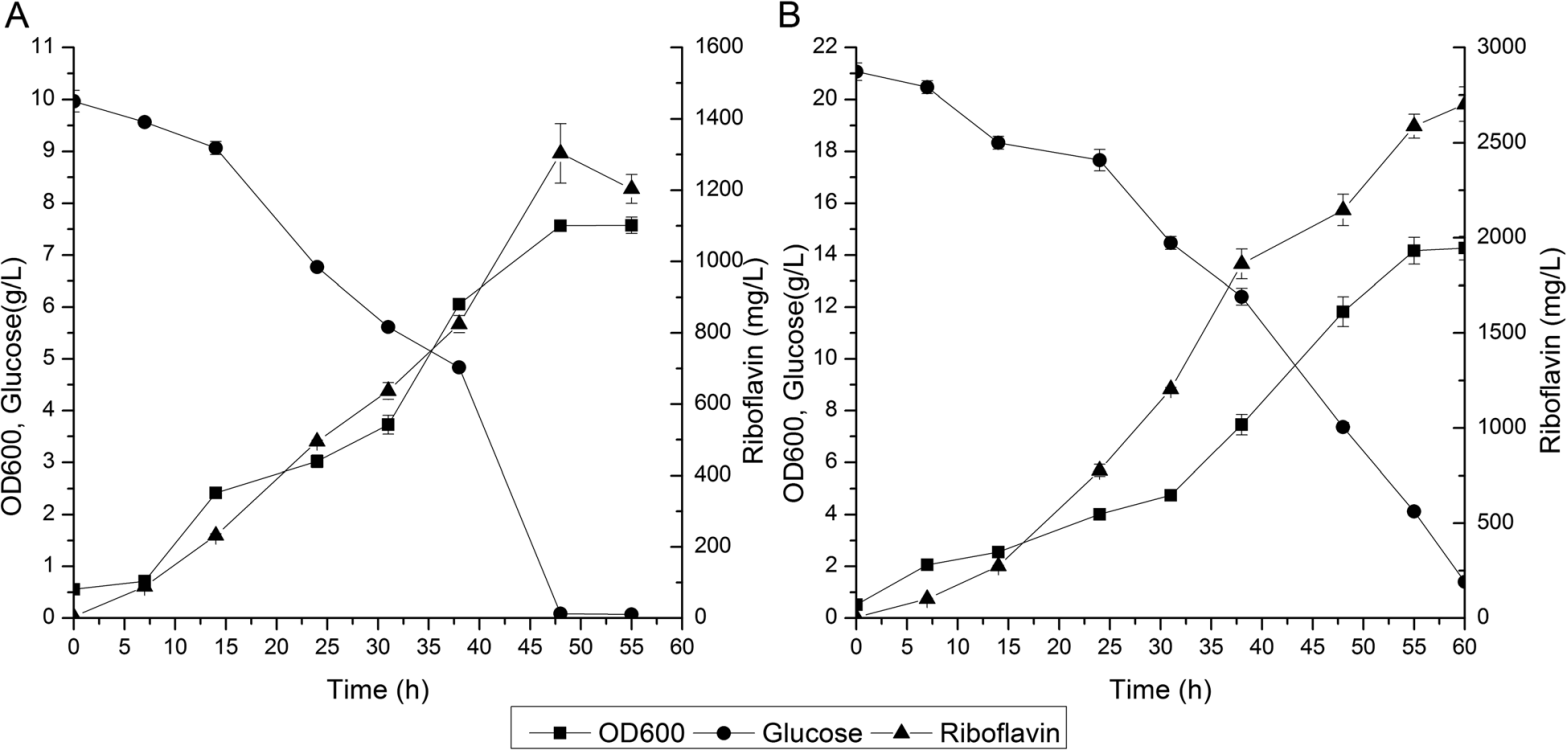
**Time-course profiles of riboflavin production, biomass accumulation and glucose consumption of RF05S-M40 in diferent mediums.** Strains were cultured in the non-optimized MS medium **(A)** and optimized MS medium **(B)**. Symbols represent the mean values for each parameter ± standard deviation of measurements from three independent experiments.

The time profiles of cell growth, glucose consumption and riboflavin production in the non-optimized medium and the optimized medium were shown in Figure 5. A maximal cell density was attained and residual glucose was almost exhausted after 60 h culture. A maximal riboflavin production reached 2702.8±89.9 mg/L using the optimized medium with a yield of 137.5 mg riboflavin/g glucose in shake flask cultivation, which was 2-fold higher than that of non-optimized medium. The final titer and specific production rates of RF05S-M30 were still lower than that of the industrial producers such as *A. gossypii*, *C. famata*, *B. subtilis*, and *Corynebacterium ammoniagenes*, which up to 20 g/L riboflavin can be reached with specific production rates of 20.7 mg/g CDW/h in industrial fed-batch production processes [[5],[13],[47]]. However, the yields of these industrial producers were lower than that of the recombinant *E. coli* (e.g. 0.138 for RF05S-M40 versus 0.100 g riboflavin/g glucose for *C. famata* [[22],[47]]). In addition, the specific glucose uptake rate of RF05S-M40 was still very low. Therefore, improving the glucose uptake rate could be an effective approach to further improving riboflavin production in RF05S-M40. Moreover, several published reports have shown that enhancing the availability of GTP increased the production of in *B. subtilis* and *A. gossypii* [[16],[17],[48],[49]], and thus further engineering the purine pathway might be a possible target for enhancing riboflavin production. In addition, compared with previous studies [[47]], the fermentation strategy used in our research is much rougher. The riboflavin titer and productivity of the strain constructed in this work could be further increased by fed-batch fermentation optimization.

## Conclusions

For the first time, we constructed a genetically defined riboflavin producing *E. coli* strain. Riboflavin synthetic pathway, PP pathway, central metabolic pathways, and riboflavin consumption pathway were systematically engineered for direct and efficient conversion of glucose to riboflavin. The *E. coli* strain RF05S-M40 has the highest yield among all reported riboflavin production strains in shake flask culture. This work also demonstrates that *E. coli* has a potential to be a host for riboflavin bioproduction.

## Methods

### Strain, medium, and cultivation condition

*E. coli* K-12 MG1655 (Coli Genetic Stock Center) was selected for engineering of the basic riboflavin-producing strains using a unique gene manipulate method. All strains used in this study are summarized in Table 5. *E. coli* DH5α was used for the propagation of vector DNA. During strain construction, cultures were grown at 30°C or 37°C in Luria-Bertani (LB) broth (per liter: 10 g tryptone, 5 g yeast extract, 10 g sodium chloride) and supplemented with antibiotics as appropriate.

**Table 5:** Strains used in this study

**Strain**	**Characteristics**	**Source**
*Escherichia coli* DH5α	Coli Genetic Stock Center strain (CGSC) No. 12384	CGSC^a^
*E. coli* MG1655	Coli Genetic Stock Center strain (CGSC) No. 7740	CGSC
*Bacillus subtilis* 168	Wild type	Lab collection
RF01S	*E. coli* MG1655 containing p20C-EC10	This study
RF02	*E. coli* MG1655, *Δpgi*	This study
RF02S	RF02containing p20C-EC10	This study
RF03	*E. coli* MG1655, *Δpgi, Δedd, Δeda*	This study
RF03S	RF03 containing p20C-EC10	This study
RF05	*E. coli* MG1655, *Δpgi, Δedd, Δeda*, P*trc*-*acs*	This study
RF05S	RF05 containing p20C-EC10	This study

^a^Coli Genetic Stock Center.

### Plasmid construction

For the construction of p5C-EC10, p15C-EC10 and p20C-EC10 plasmids, structural genes, including *ribA*, *ribB, ribD, ribE,* and *ribC*, were amplified by PCR with oligonucleotide primers FuECribA-F, FuECribA-R, FuECribB-F, FuECribB-R, FuECribD-F, FuECribD-R, FuECribE-F, FuECribE-R, FuECribC-F, and FuECribC-R using the genomic DNA of *E. coli* MG1655 as templates. The five fragments, were spliced by overlapping extension (SOE)-PCR [[50]], constructing *E. coli rib* operon EC10, which was then cloned into p5C, p15C, p20C between the EcoRI and HindIII restriction sites. A fragment BSrib containing the riboflavin operon from *B. subtilis* was amplified with oligonucleotide primers BSrib-F/BSrib-R and cloned into p20C between the EcoRI and BamHI restriction sites, creating plasmid p20C-BSrib. To construct p20C-BS15 plasmid, structural genes, including BD*ribA,* BS*ribD,* BS*ribH,* BS*ribE*, were amplified by PCR with oligonucleotide primers FuBSribA-F, FuBSribA-R, FuBSribD-F, FuBSribD-R, FuBSribH-F, FuBSribH-R, FuBSribE-F and FuBSribE-R using the genomic DNA of *B. subtilis* 168 as templates. The four fragments were spliced by SOE-PCR, constructing BS15. This was then cloned into p20C, obtaining the expressing plasmid p20C-BS15. Plasmids used were listed in Table 6.

**Table 6:** Plasmids used in this study

**Plasmid**	**Characteristics**^**a**^	**Source**
pUC18-SVG	pUC18 P_vegI_-*gnd*361, Amp^r^, Spc^r^	[[13]]
pUC18-SVZ	pUC18 P_vegI_-*zwf*243, Amp^r^, Spc^r^	[[13]]
p5C	Expression vector, pSC101 replication, Ptrc, Amp^r^	Lab collection
p15C	Expression vector, p15A replication, Ptrc, Amp^r^	Lab collection
p20C	Expression vector, pBR322 replication, Ptrc, Amp^r^	Lab collection
p5C-EC10	pSC101 replication, Amp^r^, P_trc_-synRBS-*ribA*-synRBS-*ribB*- synRBS-*ribD*-synRBS-*ribE*-synRBS-*ribC*	This study
p15C-EC10	p15A replication, Amp^r^, P_trc_-synRBS-*ribA*-synRBS-*ribB*-synRBS- *ribD*-synRBS-*ribE*-synRBS-*ribC*	This study
p20C-EC10	pBR322 replication, Amp^r^, P_trc_-synRBS-*ribA*-synRBS-*ribB*- synRBS-*ribD*-synRBS-*ribE*-synRBS-*ribC*	This study
p20C-BS15	pBR322 replication, Amp^r^, P_trc_-synRBS-*ribB/A*-synRBS-*ribD*- synRBS-*ribE*-synRBS-*ribC*	This study
p20C-BSrib	pBR322 replication, Amp^r^, P_trc_-BSrib operon	This study
p15Trc	Expression vector, p15A replication, Cm^r^	Lab collection
p15Trc -*zg*	p15A replication, Cm^r^, P_trc_-synRBS-*zwf*-synRBS-*gnd*, *zwf* and *gnd* genes from *C. glutamicum*	This study
p15Trc -*zgp*	p15A replication, Cm^r^, P_trc_-synRBS-*zwf*-synRBS-*gnd*-synRBS-*pgl*, *zwf* and *gnd* genes from *C. glutamicum*, *pgl* gene from *E. coli*	This study
pTKS/CS	p15A replication, Cm^r^, Tet^r^, I-SceI restriction sites	[[52]]
pTKRED	pSC101 replication, temperature sensitive replication origin, Spc^r^, P_araBAD_-driven I-SceI gene, Red recombinase expression plasmid, lac-inducible expression	[[52]]

^a^*Abbreviations*: *Amp* ampicillin, *Cm* chloramphenicol, *Km* kanamycin, *Tet* tetracycline, *r* resistance.

For the construction of p15Trc-*zg*, *zwf*^(A243T)^ and *gnd*^(S361F)^ from *C. glutamicum* were firstly cloned in a p15A origin vector under the control of Ptrc individually by CPEC [[51]] with primer sets p15Trc-1BF, p15Trc-1BR, p15Trc-zwfF, p15Trc-zwfR, p15Trc-gndF, p15Trc-gndR respectively. Then, the *zwf*^(A243T)^ and *gnd*^(S361F)^ genes from *C. glutamicum* and *pgl* gene from *E. coli* were assembled into a p15A origin vector under the control of Ptrc by CPEC, obtaining plasmid p15Trc-*zgp*. All primers were listed in Additional file 1: Table S1, and plasmids constructed were listed in Table 6.

### Genome engineering: gene deletion, replacement, and insertion

**Figure 6:**
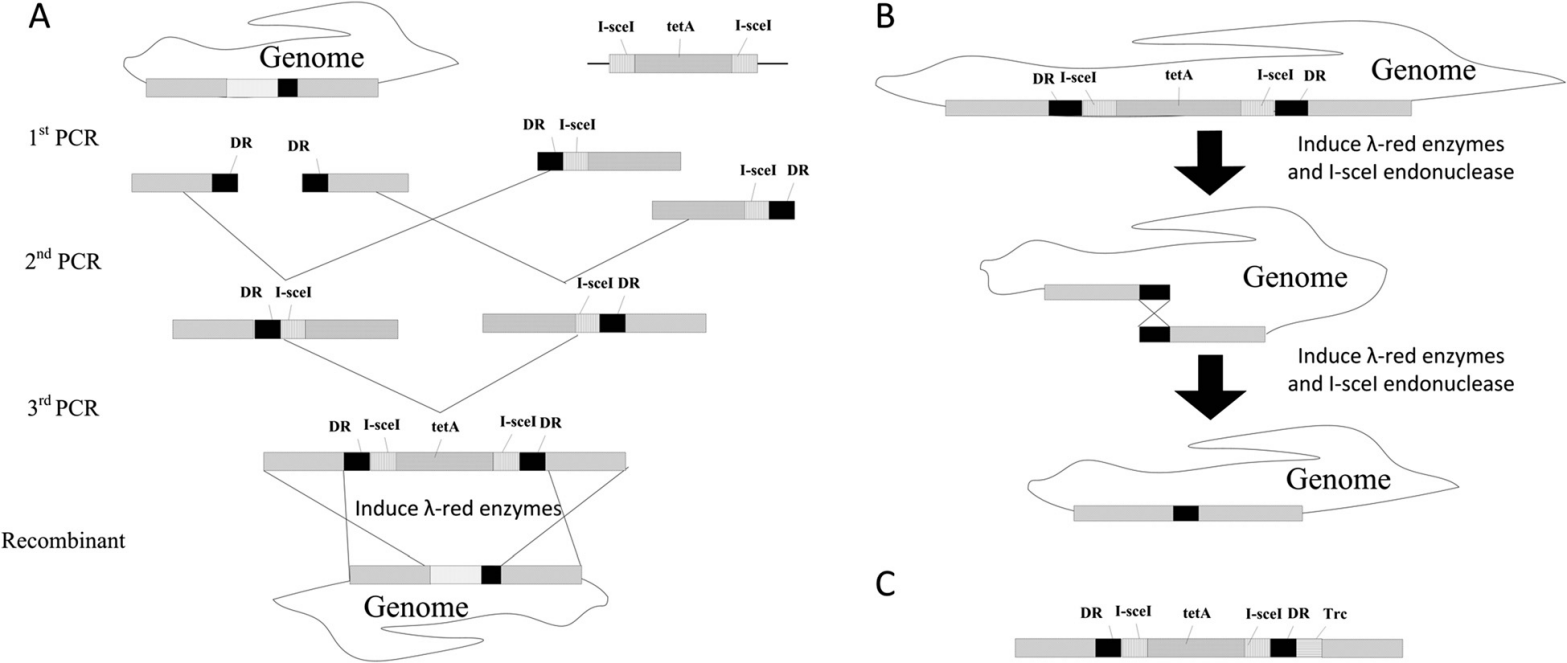
**Strategies for scarless chromosomal gene deletion, promoter replacement and RBS fine-tuning. (A)** Genome editing cassettes are constructed by three rounds of PCR and recombinants after the first round of recombineering were selected by Tet^r^. **(B)** In the second step, the TetA marker were released by simultaneous induction of I-SceI and Red recombinase expression. **(C)** The genetic construct for promoter replacement after the first round of recombination. DR for duplicate region; I-sceI, I-SceI endonuclease recognition site.

The strategy of fragment construction and genome manipulation is outlined in Figure 6. The fragments were amplified in three rounds of PCR. In the first round of PCR (Figure 6A), the flanks and the selectable marker are amplified. *E. coli* chromosome was used as a PCR template to amplify the 5′ flank and the 3′ flank of the target gene. Primers U-F/U-R and L-R/L-F were used to amplify the 5' homologous arm and the 3' homologous arm, respectively. The overlapping marker fragments "tetA-U" and "tetA-L" of the *tetA* gene were amplified from pTKS/CS using T2/T-F and T1/T-R primers, respectively (Additional file 1: Table S1). The 5′extensions for primers U-R and L-F, containing I-sceI recognition site and 30 bp DR sequence, were complementary to the T-F and T-R primer sequences, respectively. The length of the homologous arms was 250–500 bp as indicated. The second round of PCR, the 5' homologous arm and tet-U, the 3' homologous arm and tet-L were spliced by SOE-PCR, using primer U-F/T2 and T1/L-R, respectively. The final fragment was constructed using SOE-PCR with primer U-F/L-R and electroporated into target cells carrying pTKRED, according to the method of Kuhlman [[52]]. Firstly, a linear fragment consisting of *tetA* cassette flank by duplication regions (DRs) and I-SceI recognition sites was used to replace or insert into target site for obtaining an intermediate strain. The tetracycline resistance mutants were selected and confirmed by PCR. Then, arabinose was added to induce I-SceI endonuclease expression for cleaving the *tetA* and facilitating the recombination between DRs. The loss of *tetA* in the desired recombinant strains were selected on L-arabinose plates and verified by PCR. This method was employed to delete the *pgi*, *edd*, *eda*, insert *trc* promoter for *acs* gene, and modulate the expression of *ribF*. The primers used during construction are listed in Additional file 1: Table S1.

As mentioned above, the strategy of fragment construction and genome manipulation was used to modulate the expression of *ribF*. The sequence of primer ribF L_F is *TACAAGGTATACTCGGACGATTTTCACTGT*HKTGWRCCAGMC**ATG**. The RBS library of *ribF*, design with RBS Calculator [[42]], was placed between the DR sequence (italicized) and the start codon of *ribF* (bold). The primers used during construction were listed in Additional file 1: Table S1.

### Quantitative real-time reverse transcription (RT)-PCR analysis

The recombinant strains were cultured in LB medium with 1% glucose. Cells were harvested when OD600 reached 1. Total mRNA were extracted using the RNAprep pure Cell/Bacteria Kit (Tiangen, Beijing, China) as described by the manufacturer. The cDNA was amplified using FastQuant RT Kit (Tiangen, Beijing, China) with the total mRNA as the templates. Samples were then analyzed using a Light Cycler®480 II (Roche, Basel, Switzerland) with RealMasterMix (SYBR Green I) (Tiangen, Beijing, China). Quantity real-time PCR amplification primers were listed in Additional file 1: Table S1. The *rrsA* gene was selected as internal standard for normalization and three biological replicates were performed. The obtained data were analyzed by using the 2^-ΔΔCt^ method described previously [[53]].

### Enzyme assay and preparation of cell extracts

The engineering strains were cultivated in LB medium containing 1% glucose. They were cultured to mid-exponential phase and the cells were harvested by centrifugation for 10 min at 12000×g and 4°C, washed twice with 100 mM potassium phosphate (pH 7.5)-1 mM dithiothreitol-0.1 mM EDTA. The cells were resuspended and sonicated for 5 min in an ice bath (130 W, 20 kHz, pulse: 5 s on; 5 s off). After centrifugation (13,000×g, 10 min, 4°C), the supernatants were used as cell extracts. When necessary, IPTG was used at a concentration of 2 mM for induction. The activities of GTP cyclohydrolase II, riboflavin synthase and flavokinase activity were measured as previously described [[54]–[56]]. The activities of glucose 6-phosphate-1-dehydrogenase and 6-phosphogluconate dehydrogenase were determined as previously described [[57]]. Total protein concentrations were determined by the Bradford method using bovine serum albumin as standard [[58]].

### Production of riboflavin from glucose

For riboflavin production, LBG medium (LB medium with 1% (W/V) glucose), modified M9 medium, or modified minimal salt (MS) medium was selected. An amount of 20 g/liter glucose or 10 g/liter glucose was added as the carbon source except as indicated. The modified M9 medium contained (per liter) 6 g Na_2_HPO_4_, 3 g KH_2_PO_4_, 1 g NH_4_Cl, 0.5 g NaCl, 2 mM MgSO_4_, 0.1 mM CaCl_2_, and yeast extract as indicated. The MS medium contained (per liter) 3.8 g Na_2_HPO_4_, 1.5 g KH_2_PO_4_, 1.0 g (NH_4_)_2_SO_4_, 0.2 g MgSO_4_, yeast extract as indicated, and 2% (v/v) trace element solution as described in previous studies [[59]].

For fermentation, transformed strains were cultured overnight in 5 ml LB media at 37°C, then transferred into 50 ml LB media with 1% (v/v) of the seed culture. This was cultured to mid-exponential phase at 240 rpm, 37°C, and frozen at −80°C in 15% glycerol (v/v). The inoculum was prepared from frozen seed stocks in LB medium supplemented with 100 mg/L ampicillin, grown to mid-exponential phase, and then used to inoculate batch cultures with 1% (v/v) of the seed culture, at 37°C or 31°C as indicated. When appropriate, IPTG was used at a concentration of 2 mM for induction. For each strain, three parallel fermentations were performed.

### Analytical methods

Cell concentration was calculated from OD 600 nm measurements. Glucose concentration was measured enzymatically using a glucose analyzer (Model-SBA40, Shandong, China). To determine the concentration of acetate, culture samples were centrifuged at 12,000×g for 5 min and the aqueous supernatant used for HPLC analysis on an Agilent 1100 Series HPLC system equipped with an Aminex HPX-87H anion exchange column (Bio-Rad Laboratories, Richmond, CA, USA) and refractive index detector. The column was eluted with 5 mM sulfuric acid at a flow rate of 0.4 ml/min. Standards were prepared for acetate for both the refractive index detector, and calibration curves were created. For riboflavin measurements, culture samples were diluted with 0.05 M NaOH to the linear range of the spectrophotometer and the A444 was immediately measured. The results represented the means±S.D. of three independent experiments. Dry cell weight (DCW) was calculated from the optical density at 600 nm (1 OD_600_=0.38 g DCW/L).

## Competing interests

The authors declare that they have no competing interests.

## Authors’ contributions

ZQL designed the experiments; ZQL, ZWW, ZBX, and YFL performed the experiments; ZQL, ZWW and TC wrote the manuscript; TC and XMZ supervised the work; and all authors contributed to the discussion of the research. All authors read and approved the final manuscript.

## Additional file

## Supplementary Material

Additional file 1: Table S1. Primers used in this study. **Table S2.** Experimental design for evaluating factors influencing biomass and riboflavin production. Click here for file
