# Supplementary material for: Metabolic engineering of Escherichia coli for the production of riboflavin
Source: Microb Cell Fact. 2014 Jul 16;13:104. doi: 10.1186/s12934-014-0104-5 (PMC4223517; doi:10.1186/s12934-014-0104-5)
Supplement: Additional file 1: Table S1. — Primers used in this study. Table S2. Experimental design for evaluating factors influencing biomass and riboflavin production. [file s12934-014-0104-5-S1.docx]

**Additional file 1**

**Table S1. Primers used in this study**

| Primer name | Primer sequencea |
| --- | --- |
| FuECribA-F | CGGAATTCAAGTCCTCAACTACCAAGGAGAAAACAATGCAGCTTAAACGTGTGGCA |
| FuECribA-R | TGATTCATTAAGTTAGGGCCCCCTTATTTTATAGATTTATTTGTTCAGCAAATGGCCC |
| FuECribB-F | CAAATAAATCTATAAAATAAGGGGGCCCTAACTTAatgAATCAGACGCTACTTTCCTC |
| FuECribB-R | ACTGTGTTTTCCTCCTTGTCTATCTACGGGATCCTCAGCTGGCTTTACGCTCATGTG |
| FuECribD-F | GAGGATCCCGTAGATAGACAAGGAGGAAAACACAGTGCAGGACGAGTATTACATG |
| FuECribD-R | TATTTCCTCCTCTTCCTGCGTTTGACGTATGTGTCGACTCATGCACCCACTAAATGCAG |
| FuECribE-F | GTCGACACATACGTCAAACGCAGGAAGAGGAGGAAATACATGAACATTATTGAAGCTA |
| FuECribE-R | ATCTCTCTCCTCCTGTAAGAGGCGCGTCTGCGTCCTCTCAGGCCTTGATGGCTTTC |
| FuECribC-F | GAGGACGCAGACGCGCCTCTTACAGGAGGAGAGAGATGTTTACGGGGATTGTACAG |
| FuECribC-R | AGCCAAGCTTTCAGGCTTCTGTGCCTGGT |
| BSrib-F | GCGCGAATTCTACTAGAGATTAAAGAGGAGAAAATGGAAGAGTATTATATGAAGCTGGCC |
| BSrib-R | CGCGGATCCTTATTCAAATGAGCGGTTTAAATTTG |
| FuBSribA-F | CGGAATTCGAACCGCACTAAGGAGGTACTACAAACGGAATGTTTCATCCGATAGAAGA |
| FuBSribA-R | TCCTCCTGGGGTCTCCGCTCTAAGTGGAGGGTACCTTAGAAATGAAGTAAATGACCT |
| FuBSribD-F | TAAGGTACCCTCCACTTAGAGCGGAGACCCCAGGAGGAACCATGGAAGAGTATTATATG |
| FuBSribD-R | GTATCCCTCCTTAAGACGTCGGCGGAAACCGACGGATCCCTATTCCTTTGTCGGTTTTG |
| FuBSribH-F | GGGATCCGTCGGTTTCCGCCGACGTCTTAAGGAGGGATACGCATGAATATCATACAAGG |
| FuBSribH-R | TGCTCTAGAGCTTACCATGGTTATTCAAATGAGCGGTTTA |
| FuBSribE-F | TAACCATGGGGACCAAGATCCACTCTAGACTAAGGAGGAAGTATGTTTACAGGAATTAT |
| FuBSribE-R | TGCTCTAGACTAAAAGCCGTTTTCGCTTA |
| Primers for p15Trc-zg | |
| p15Trc-1BF | CCGAGGTTGAAGCTTAAGTCGACCTGCAGGCATGCAAGCT |
| p15Trc-1BR | ACACATTATACGAGCCGGAAGCATAAAGTGTAAACAGCTCATTTCAGA |
| p15Trc-zwfF | TCTGAAATGAGCTGTTTACACTTTATGCTTCCGGCTCGTATAATGTGT |
| p15Trc-zwfR | TGACGGCATATATTATCTCCTTATCCTTGGTCGGCTTATGGCCTGCGCCAGGTGT |
| p15Trc-gndF | AGGCCATAAGCCGACCAAGGATAAGGAGATAATATATGCCGTCAAGTACGATC |
| p15Trc-gndR | AGCTTGCATGCCTGCAGGTCGACTTAAGCTTCAACCTCGG |
| Primers for p15Trc-zgp | |
| p15Trc-3BF | GGTGGTTAACGCACACTAAGTCGACCTGCAGGCATGCAA |
| p15Trc-3BR | TCACCTGTTAACCTCCTTAATGTAAAAGTAGTATCGGGTACCGAGCTCGAATTC |
| p15Trc-301F | GTACCCGATACTACTTTTACATTAAGGAGGTTAACAGGTGAGCACAAACACGACC |
| p15Trc-301R | TTGCTTCATGTGGTACTCTCTCTTACTAGGGGTGTTAAGCTTCAACCTCGGAG |
| p15Trc-pglF | AAGCTTAACACCCCTAGTAAGAGAGAGTACCACATGAAGCAAACAGTTTATATCGC |
| p15Trc-pglR | TTGCATGCCTGCAGGTCGACTTAGTGTGCGTTAACCACC |
| Primers for genetic manipulation | |
| Δpgi U_F | AGCCGTTCAATCACCTCG |
| Δpgi U_R | ACATTACCCTGTTATCCCTATAGCAATACTCTTCTGATTTTGAG |
| Δpgi T_F | GAAGAGTATTGCTATAGGGATAACAGGGTAATGTACCATTTAC |
| Δpgi T_R | TAGCAATACTCTTCTGATTTTGAGAATTGTATTACCCTGTTATCCCTACTAAGC |
| Δpgi L_F | ACAATTCTCAAAATCAGAAGAGTATTGCTATCATCGTCGATATGTAGGCCG |
| Δpgi L_R | ATTAATCTGCGTATTTTCCGAA |
| ΔED U_F | AACCTGCAAATCACCAAGCTG |
| ΔED U_R | TAGTCGGGCAGAAACGGACCTGGGAGAACGGACCTCGTGGGTGAATCCGCA |
| ΔED T_F | TCCGTTCTCCCAGGTCCGTTTCTGCCCGACTAGGGATAACAGGGTAATGTACC |
| ΔED T_R | GTCGGGCAGAAACGGACCTGGGAGAACGGAATTACCCTGTTATCCCTACTAA |
| ΔED L_F | TAGGGATAACAGGGTAATTCCGTTCTCCCAGGTCCGT |
| ΔED L_R | CAGAGAGTGTTGTTGACGCCAT |
| ribF U_F | CTTTGTCGCCAGCTTCGAT |
| ribF U_R | CTCGGACGATTTTCACTGTATTACCCTGTTATCCCTACTAAGCAC |
| ribF T_F | GTGCTTAGTAGGGATAACAGGGTAATACAGTGAAAATCGTCCGAG |
| ribF T_R | ACAGTGAAAATCGTCCGAGTATACCTTGTATAGGGATAACAGGGTAATGTACCATTTAC |
| ribF L_F | TACAAGGTATACTCGGACGATTTTCACTGTHKTGWRCCAGMCATGAAGCTGATACGCGG |
| ribF L_R | TTACGCGCAAATGCTTCACC |
| Pacs U_F | TTCACGACAGTAACCGCACCT |
| Pacs U_R | ACATTATACGAGCCGGATGATTAATTGTCAACAGCTCTGTAGGGGTATTGGCAGTTTTG |
| Pacs T_F | GTTGACAATTAATCATCCGGCTCGTATAATGTGTGGTAGGGATAACAGGGTAATGTACC |
| Pacs T_R | CCACACATTATACGAGCCGGATGATTAATTGTCAAATTACCCTGTTATCCCTACTAAG |
| Pacs L_F | ACAATTAATCATCCGGCTCGTATAATGTGTGGTTTAACGCTTATGCCACATATTA |
| Pacs L_R | GTGCCGTCCTCGTACCAT |
| T1 | GCGTGAAGTGGTTCGGTTG |
| T2 | CTGTAATGCAGGTAAAGCGATC |
| Primers for RT-PCR | |
| RTrrsA_F | TACGACCAGGGCTACACACG |
| RTrrsA_R | ATCCGGACTACGACGCACTT |
| RTedd_F | CGGTAATGGTAATGAATGGA |
| RTedd_R | ATGGTGTGGTTAGTGGAA |
| RTeda_F | CACAGTTCGCAATTAGCC |
| RTeda_R | CGGAATCAGAGGAATAGTC |
| RTacs_F | GACCTACTTCTCCACCTTCA |
| RTacs_R | CGGTTATCCAGTAATAGCCATC |

^a^Underlined letters are restriction enzyme cut sites.

**Table S2. Experimental design for evaluating factors influencing biomass and riboflavin production.**

| Run | X1 | X2 | X3 | X4 | X5 | X6 | X7 | X8 | X9 | X10 | X11 | X12 | Riboflavin (mg/L) |
| --- | --- | --- | --- | --- | --- | --- | --- | --- | --- | --- | --- | --- | --- |
| 1 | 10 | 5 | 1 | 0.2 | 4.78 | 1.875 | 2 | 0 | 0 | 0 | 0 | 0 | 1303.69$\pm$83.35^a^ |
| 2 | 10 | 5 | 0.5 | 0.1 | 4.78 | 1.875 | 2 | 0 | 0 | 0 | 0 | 0 | 1164.61$\pm$27.79 |
| 3 | 10 | 5 | 1 | 0.2 | 2.39 | 0.938 | 2 | 0 | 0 | 0 | 0 | 0 | 1063.84$\pm$58.57 |
| 4 | 10 | 5 | 1 | 0.2 | 4.78 | 1.875 | 1 | 0 | 0 | 0 | 0 | 0 | 1283.57$\pm$51.73 |
| 5 | 10 | 5 | 1.5 | 0.3 | 4.78 | 1.875 | 2 | 0 | 0 | 0 | 0 | 0 | 1274.10$\pm$69.52 |
| 6 | 10 | 5 | 1 | 0.2 | 7.17 | 2.814 | 2 | 0 | 0 | 0 | 0 | 0 | 1312.56$\pm$52.53 |
| 7 | 10 | 5 | 1 | 0.2 | 4.78 | 1.875 | 3 | 0 | 0 | 0 | 0 | 0 | 1279.25$\pm$39.13 |
| 8 | 20 | 5 | 1 | 0.2 | 4.78 | 1.875 | 2 | 0 | 0 | 0 | 0 | 0 | 1653.91$\pm$77.16 |
| 9 | 10 | 5 | 1 | 0.2 | 7.17 | 2.814 | 3 | 0 | 0 | 0 | 0 | 0 | 1278.82$\pm$80.36 |
| 10 | 20 | 5 | 1 | 0.2 | 7.17 | 2.814 | 3 | 0 | 0 | 0 | 0 | 0 | 1467.39$\pm$33.99 |
| 11 | 20 | 5 | 1 | 0.2 | 7.17 | 2.814 | 3 | 1 | 0 | 0 | 0 | 0 | 1434.99$\pm$58.80 |
| 12 | 20 | 5 | 1 | 0.2 | 7.17 | 2.814 | 3 | 2 | 0 | 0 | 0 | 0 | 1365.11$\pm$72.92 |
| 13 | 20 | 5 | 1 | 0.2 | 7.17 | 2.814 | 3 | 3 | 0 | 0 | 0 | 0 | 1372.07$\pm$10.87 |
| 14 | 20 | 5 | 1 | 0.2 | 7.17 | 2.814 | 3 | 0 | 1 | 0 | 0 | 0 | 2000.93$\pm$5.11 |
| 15 | 20 | 5 | 1 | 0.2 | 7.17 | 2.814 | 3 | 0 | 2 | 0 | 0 | 0 | 2114.12$\pm$15.63 |
| 16 | 20 | 5 | 1 | 0.2 | 7.17 | 2.814 | 3 | 1 | 1 | 0 | 0 | 0 | 1628.87$\pm$33.85 |
| 17 | 20 | 5 | 1 | 0.2 | 7.17 | 2.814 | 3 | 2 | 2 | 0 | 0 | 0 | 2078.40$\pm$40.70 |
| 18 | 20 | 5 | 1 | 0.2 | 7.17 | 2.814 | 3 | 1 | 2 | 0 | 0 | 0 | 2542.68$\pm$68.64 |
| 19 | 20 | 5 | 1 | 0.2 | 7.17 | 2.814 | 3 | 1 | 3 | 0 | 0 | 0 | 2339.88$\pm$91.86 |
| 20 | 20 | 5 | 1 | 0.2 | 7.17 | 2.814 | 3 | 2 | 1 | 0 | 0 | 0 | 2115.58$\pm$46.07 |
| 21 | 20 | 5 | 1 | 0.2 | 7.17 | 2.814 | 3 | 3 | 1 | 0 | 0 | 0 | 2241.64$\pm$71.32 |
| 22 | 20 | 5 | 1 | 0.2 | 7.17 | 2.814 | 3 | 2 | 1 | 0 | 0 | 0 | 1773.10$\pm$71.33 |
| 23 | 20 | 5 | 1 | 0.2 | 7.17 | 2.814 | 4 | 2 | 1 | 0 | 0 | 0 | 1693.98$\pm$90.55 |
| 24 | 20 | 5 | 1 | 0.2 | 7.17 | 2.814 | 3 | 1 | 2 | 0.5 | 0 | 0 | 1807.74$\pm$62.43 |
| 25 | 20 | 5 | 1 | 0.2 | 7.17 | 2.814 | 3 | 1 | 2 | 0 | 0.5 |  | 1561.63$\pm$59.13 |
| 26 | 20 | 5 | 1 | 0.2 | 7.17 | 2.814 | 3 | 1 | 2 | 0 | 0 | 1 | 1334.84$\pm$75.65 |
| 27 | 20 | 5 | 1 | 0.2 | 7.17 | 2.814 | 3 | 1 | 2 | 0.5 | 0.5 | 0 | 2069.11$\pm$96.34 |
| 28 | 20 | 5 | 1 | 0.2 | 7.17 | 2.814 | 3 | 1 | 2 | 0.5 | 0 | 1 | 1640.97$\pm$26.61 |
| 29 | 20 | 5 | 1 | 0.2 | 7.17 | 2.814 | 3 | 1 | 2 | 0 | 0.5 | 1 | 1492.58$\pm$91.29 |
| 30 | 20 | 5 | 1 | 0.2 | 7.17 | 2.814 | 3 | 1 | 2 | 0.5 | 0.5 | 1 | 2193.30$\pm$74.43 |
| 31 | 20 | 5 | 1 | 0.2 | 7.17 | 2.814 | 3 | 1 | 2 | 0.5 | 1 | 1 | 1891.80$\pm$69.83 |
| 323 | 20 | 5 | 1 | 0.2 | 7.17 | 2.814 | 3 | 1 | 2 | 1 | 0.5 | 1 | 1960.12$\pm$47.18 |
| 33 | 20 | 5 | 1 | 0.2 | 7.17 | 2.814 | 3 | 1 | 2 | 0 | 0.5 | 0 | 1994.18$\pm$43.24 |
| 34 | 20 | 5 | 1 | 0.2 | 7.17 | 2.814 | 3 | 1 | 2 | 1 | 1 | 0 | 2275.44$\pm$51.38 |
| 35 | 20 | 5 | 1 | 0.2 | 7.17 | 2.814 | 3 | 1 | 2 | 1 | 1 | 1 | 1941.59$\pm$78.29 |
| 36 | 20 | 5 | 1 | 0.2 | 7.17 | 2.814 | 3 | 1 | 3 | 1 | 1 | 0 | 2532.75$\pm$54.48 |
| 37 | 20 | 5 | 1 | 0.2 | 7.17 | 2.814 | 3 | 1 | 3 | 1 | 0 | 0 | 2033.02$\pm$41.87 |

^a^Samples were taken on 48h and the standard deviation was calculated from three independent samples. Cells were cultivated in LB medium containing 10 g/L glucose at 37 ˚C and 220 rpm for 48 h. X1, glucose (g/L); X2, Yeast extract (g/L); X3, (NH_4_)_2_SO_4_ (g/L); X4, MgSO_4_(g/L); X5, Na_2_HPO_4_ (g/L); X6, KH_2_PO_4_ (g/L); X7, Trace (mL); X8, Urea (g/L); X9, Glycine (g/L); X10, Glutamine (g/L); X11, Asparagine (g/L); X12, NaHCO_3_ (g/L). All datas were obtained from three parallel studies (n=3).
